# Van der Waals engineering of ferroelectric heterostructures for long-retention memory

**DOI:** 10.1038/s41467-021-21320-2

**Published:** 2021-02-17

**Authors:** Xiaowei Wang, Chao Zhu, Ya Deng, Ruihuan Duan, Jieqiong Chen, Qingsheng Zeng, Jiadong Zhou, Qundong Fu, Lu You, Song Liu, James H. Edgar, Peng Yu, Zheng Liu

**Affiliations:** 1 School of Materials Science and Engineering, Nanyang Technological University, Singapore, Singapore; 2 Jiangsu Key Laboratory of Thin Films, School of Physical Science and Technology, Soochow University, Suzhou, China; 3 Tim Taylor Department of Chemical Engineering, Durland Hall, Kansas State University, Manhattan, KS USA; 4 School of Materials Science and Engineering, State Key Laboratory of Optoelectronic Materials and Technologies, Sun Yat-sen University, Guangzhou, China; 5 CINTRA CNRS/NTU/THALES, UMI 3288, Research Techno Plaza, Singapore, Singapore; 6 Centre for Micro-/Nano-electronics (NOVITAS), School of Electrical and Electronic Engineering, Nanyang Technological University, Singapore, Singapore

**Keywords:** Information storage, Two-dimensional materials, Electronic devices

## Abstract

The limited memory retention for a ferroelectric field-effect transistor has prevented the commercialization of its nonvolatile memory potential using the commercially available ferroelectrics. Here, we show a long-retention ferroelectric transistor memory cell featuring a metal-ferroelectric-metal-insulator-semiconductor architecture built from all van der Waals single crystals. Our device exhibits 17 mV dec^−1^ operation, a memory window larger than 3.8 V, and program/erase ratio greater than 10^7^. Thanks to the trap-free interfaces and the minimized depolarization effects via van der Waals engineering, more than 10^4^ cycles endurance, a 10-year memory retention and sub-5 μs program/erase speed are achieved. A single pulse as short as 100 ns is enough for polarization reversal, and a 4-bit/cell operation of a van der Waals ferroelectric transistor is demonstrated under a 100 ns pulse train. These device characteristics suggest that van der Waals engineering is a promising direction to improve ferroelectronic memory performance and reliability for future applications.

## Abstract (web summary)

The memory retention for a ferroelectric field-effect transistor is limited by the depolarization effects and carrier charge trapping. Here, the authors fabricate a long-retention memory cell with a metal-ferroelectric-metal-insulator-semiconductor architecture built from all van der Waals single crystals.

## Introduction

Ferroelectric materials with switchable macroscopic polarization have been exploited in a wide range of technological applications such as nonvolatile memory, logic, sensors, and actuators over the past few decades^1–6^. In particular, field-effect transistors with a ferroelectric gate dielectric (FeFETs) are recognized as an attractive architecture to the existing semiconductor memory technologies owing to their nondestructive and low voltage operation, small cell size, and nonvolatility^1,2,7,8^. However, the severe depolarization effects and carrier charge trapping drastically limit the memory retention time, and prevent the commercialization of nonvolatile memory potential of FeFET using the commercially available ferroelectrics^9–11^. Furthermore, attempts to synthesize ferroelectrics on semiconductors are impeded by ferroelectric-semiconductor interdiffusion and lattice mismatch^12,13^.

The recent emerged two-dimensional (2D) van der Waals (vdW) ferroelectrics^14–16^, combined with other 2D components ranging from metals^17^, semiconductors^18^ to insulators^19^, open the possibilities for achieving a long-retention FeFET memory via vdW engineering. One of the primary advantages of vdW materials is the absence of dangling bonds, which is desirable for engineering a trap-free interface to rule out memory performance degradation^11,20^. A single-crystal vdW ferroelectric layer also affords the possibility of a square ferroelectric hysteresis loop that helps to eliminate the depolarization field induced retention loss^11,21^. Moreover, the flexibility of the vdW stacking process facilitates the engineering a desired device structure without the interdiffusion and constraints of lattice parameters.

In this work, a nonvolatile FeFET memory cell featuring a metal-ferroelectric-metal-insulator-semiconductor (MFMIS) structure is built from all vdW single crystals by integrating a ferroelectric CuInP_2_S_6_ (CIPS) layer onto the gate of a 2D FET, with MoS_2_ as the channel material, hexagonal-BN (h-BN) as the gate dielectric, and graphene as the gate contact. The vdW engineering enables the convenient integration of a single-crystal ferroelectric together with other 2D components while maintaining a clean interface. The introduction of a bipolar graphene layer into the gate stack allows the complete compensation of the ferroelectric polarization in CIPS, which is beneficial for eliminating the depolarization field. Enabled by these abilities, our vdW FeFET exhibits sub-60 mV dec^−1^ switch and a counterclockwise hysteresis loop with a memory window larger than 3.8 V and a current ratio of over 10^7^. More than 10^4^ cycles endurance, a 10-year retention, and sub-5 μs writing speed are achieved with vdW FeFET memory. Multilevel storage with 16 distinct current levels is also demonstrated in the present FeFET under a pulse train with a width of 100 ns.

## Results

**Fig. 1:**
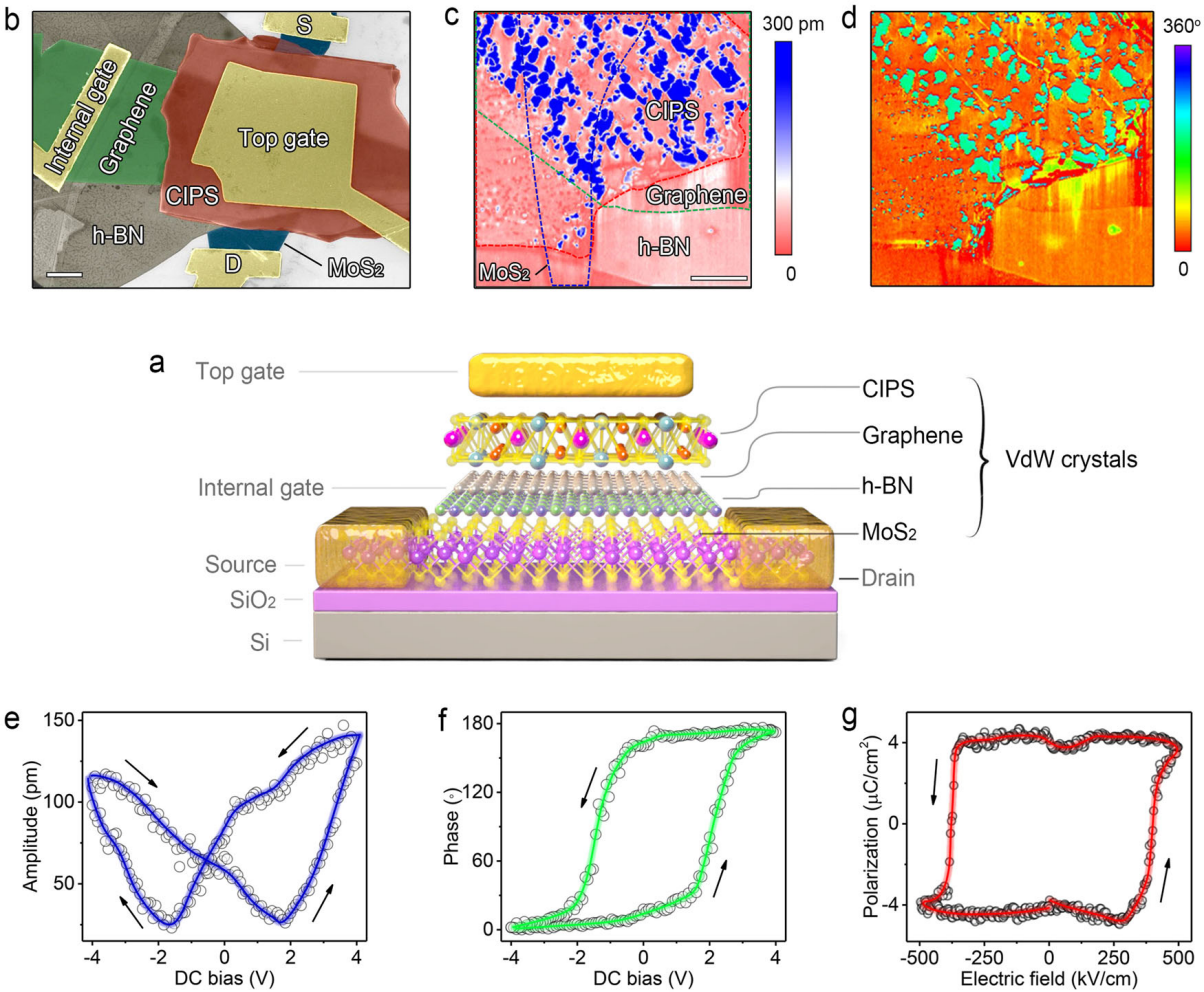
All-vdW FeFET. **a** Schematic diagram of an MoS_2_/h-BN/graphene/CIPS vdW FeFET. A ferroelectric CIPS capacitor is integrated onto the gate of the 2D FET, with MoS_2_ as the channel material, h-BN as the gate dielectric and graphene as the gate contact, to realize the vdW FeFET with an MFMIS structure. **b** False-color SEM image of a typical vdW FeFET. Scale bar, 2 μm. **c**, **d** PFM amplitude **c** and phase **d** image of an MoS_2_/h-BN/graphene/CIPS vdW heterostructure. MoS_2_, graphene, and CIPS are indicated by the blue, green, and red dashed lines, respectively. Scale bar, 2 μm. **e**, **f** The off-field PFM amplitude **e** and phase **f** hysteresis loops during the switching process for the CIPS flake. **g** The *P*−*E* hysteresis loops of a 200-nm-thick CIPS parallel-plate capacitor at 1 kHz.

Figure 1a shows the schematic structure of vdW FeFET with a metal-ferroelectric-metal-insulator-semiconductor (MFMIS) structure that comprises a CIPS ferroelectric capacitor integrated onto a 2D FET, with a few-layer MoS_2_ as the channel material, h-BN (thickness, 4−9 nm) as the gate dielectric, and graphene (thickness, 2−13 nm) as gate contact. 5 nm Cr/80 nm Au is used as the top-gate electrode and source/drain contacts. The polarization of the ferroelectric CIPS layer controls the surface potential of MoS_2,_ and the source–drain current of the underlying 2D FET. Including a bipolar graphene layer in the present device structures are crucial for compensating for both downward and upward oriented polarization in CIPS^22,23^. Additionally, the graphene layer also serves as the internal gate for characterizing the baseline 2D FET. The general layout of a vdW FeFET is given in the false-color scanning electron microscopy (SEM) image of Fig. 1b. The MoS_2_/h-BN/graphene/CIPS vdW heterostructure was created with freshly exfoliated flakes, via the dry transfer technique, before the lithography process to maintain the clear vdW interfaces^24^. Details on material preparation and device fabrication are provided in Methods and Supplementary Fig. 1.

The ferroelectricity of CIPS was characterized by vertical piezoresponse force microscopy (PFM) under dual AC resonance tracking (DART) mode and polarization versus electric field (*P*−*E*) hysteresis loop measurements. Figure 1c, d present the phase and amplitude images of local piezoresponse on a vdW heterostructure, created with a three-layer MoS_2_, 9 nm h-BN, 2 nm graphene, and 85 nm CIPS (see Supplementary Fig. 2 for the topographic image and Supplementary Fig. 3 for the determination of the layer number of MoS_2_). The bright and dark regions observed on CIPS represent the out-of-plane ferroelectric domains with upward and downward polarization, respectively. Note that the domain size of CIPS on graphene is larger than that on the h-BN or SiO_2_ substrate (see Supplementary Fig. 4 for statistics on domain size) due to the sufficient screening capability of graphene, which suggests the great potential of this vdW heterostructure for memory applications. The off-field PFM measurements were then carried out to acquire appropriate piezoresponse hysteresis loop by reducing the electrostatic effect. As presented in Fig. 1e, f, a standard butterfly amplitude loop with a 180° phase flip at the coercive voltages confirms the good ferroelectric switching nature of CIPS (see Supplementary Fig. 5 for the raw data). Polarization switching tests (Supplementary Fig. 6) were also conducted to demonstrate the out-of-plane ferroelectricity in CIPS. In the *P*−*E* measurements, voltage of a triangular waveform was applied to a CIPS parallel-plate capacitor (see Supplementary Fig. 7 for the optical image of a typical CIPS capacitor) to obtain the polarization hysteresis loops. As shown in Fig. 1g, the *P*−*E* characteristic of CIPS shows a nearly square hysteresis loop with a large (~300 kV/cm) coercive field and moderate remnant polarization (~4 μC/cm^2^) compared to ferroelectric strontium bismuth tantalite (SBT) and lead zirconium titanate (PZT)^25,26^.

**Fig. 2:**
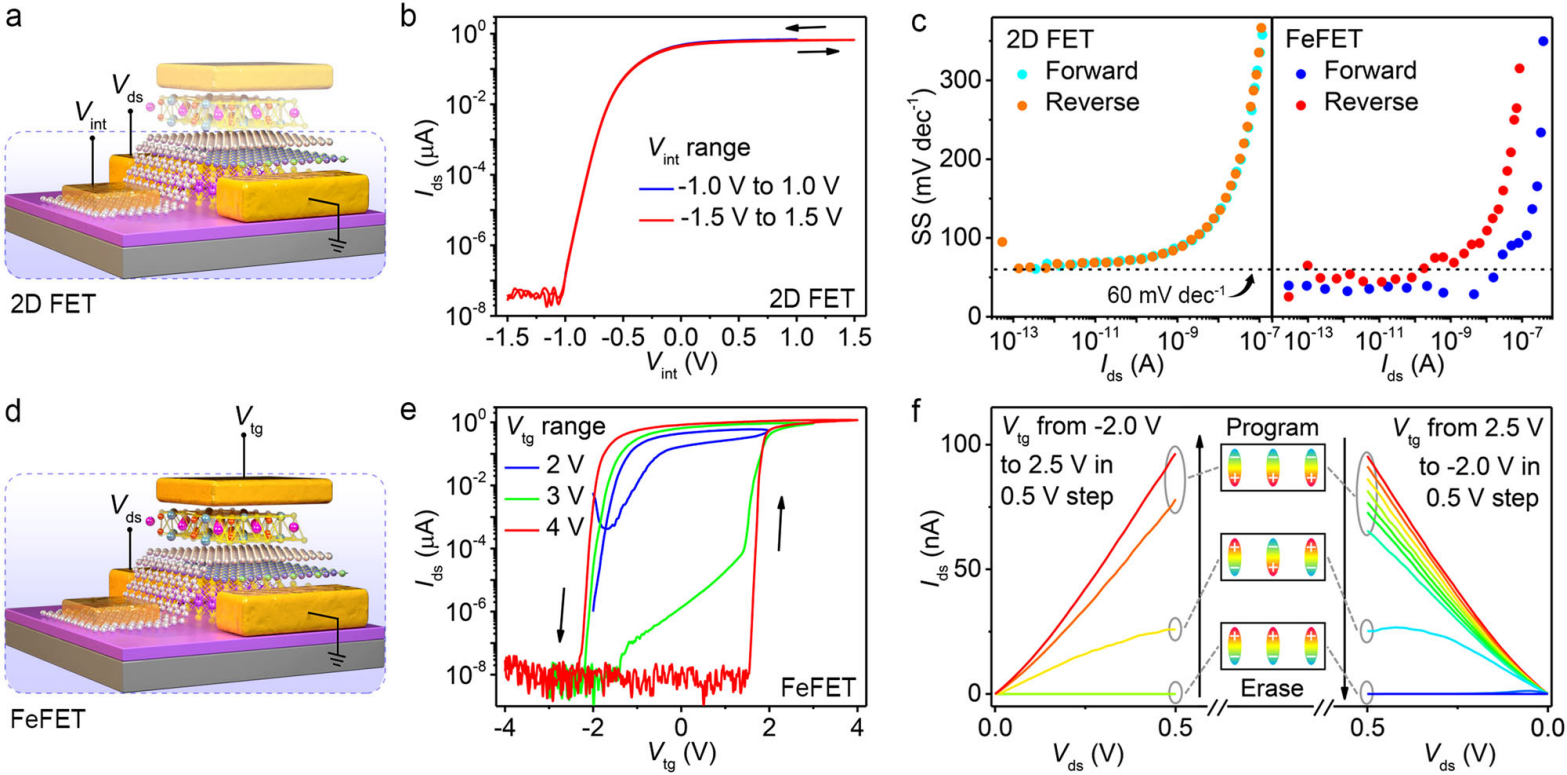
Electric transport properties of vdW FeFETs. **a** Schematics of the measurement setup used to characterize the internal 2D FET. **b** Internal-gate *I*_ds_−*V*_int_ characteristics of 2D FET at various gate voltage ranges measured with *V*_ds_ = 0.5 V and top gate floating. The device has a three-layer MoS_2_ channel and an 87-nm-thick CIPS layer. **c** Internal-gate SS−*I*_ds_ characteristics (left) and top-gate SS−*I*_ds_ characteristics (right) for the forward and reverse sweep. **d** Schematics of the measurement setup used to characterize the vdW FeFET. **e** Top-gate *I*_ds_−*V*_tg_ characteristics of the vdW FeFET at various gate voltage ranges measured with *V*_ds_ = 0.5 V and internal gate floating. **f** The *I*_ds_−*V*_ds_ curves for the forward and reverse *V*_tg_ step direction with the internal gate floating.

The room temperature electrical performance of a vdW FeFET with a three-layer MoS_2_ and 87 nm CIPS is presented in Fig. 2 (see Supplementary Fig. 8 for more vdW FeFETs with different thickness of CIPS). Figure 2a shows the schematic of the measurement setup used to characterize the underlying 2D FET through the internal gate with the top gate floating. The *I*_ds_ − *V*_int_ characteristics of 2D FET at various gate voltage ranges in Fig. 2b represent zero-hysteresis with subthreshold swing (SS) approaching the thermionic limit (Fig. 2c), indicating the absence of charge traps at the vdW interfaces^27^. The transfer curves (*I*_ds_–*V*_tg_) of the FeFET, measured using the top gate with internal gate floating (as illustrated in Fig. 2d) at different sweeping ranges of top-gate voltage, are given in Fig. 2e. The FeFET exhibits a typical *n*-type behavior with an anticlockwise hysteresis loop arising from ferroelectric polarization switching. By increasing the *V*_tg_ sweeping range, the hysteresis loop is increased to achieve a large memory window (MW) with two different stable states, namely a program state induced by remnant downward polarization, and an erase state induced by upward polarization. An MW of 3.8 V, much larger than the one of the FeFET featuring an MFS structure (Supplementary Fig. 9), with an over 10^7^ program/erase (P/E) current ratio is achieved for the vdW FeFET at the sweeping range of 4 V. Moreover, due to the negative capacitance effect in CIPS, the FeFET exhibits sub-60 mV dec^−1^ switch for both the forward and reverse sweep, with an average SS less than the Boltzmann’s limit for over 6 decades of drain current and minimum SS of 28 mV dec^−1^, as shown in Fig. 2c. The sub-60 mV dec^−1^ SS is beneficial for stabilizing P/E ratio and MW due to low off-state leakage^28^. The lowest SS demonstrated in a vdW FeFET is 17 mV dec^−1^ (Supplementary Fig. 10). Compared with an MoS_2_ based FeFET with an MFS or MFIS structure^22,29^, the improved SS in the vdW FeFET with an MFMIS structure is attributed to the sufficient compensating ability of graphene for downward and upward polarization of CIPS. The output curves (*I*_ds_ − *V*_ds_) for both the forward and reverse *V*_tg_ step direction of another vdW FeFET with a coercive voltage of ~1.2 V (see Supplementary Fig. 11 for the transfer curves) are presented in Fig. 2f. After poling CIPS up (down), the drain current remains at a low (high) level and changes slightly with an increase (decrease) of *V*_tg_ as long as *V*_tg_ does not exceed the positive (negative) coercive voltage. Owing to negative drain-induced-barrier-lowering effect in the vdW FeFET, negative differential resistance is also observed in Fig. 2f for the reverse *V*_tg_ step.

**Fig. 3:**
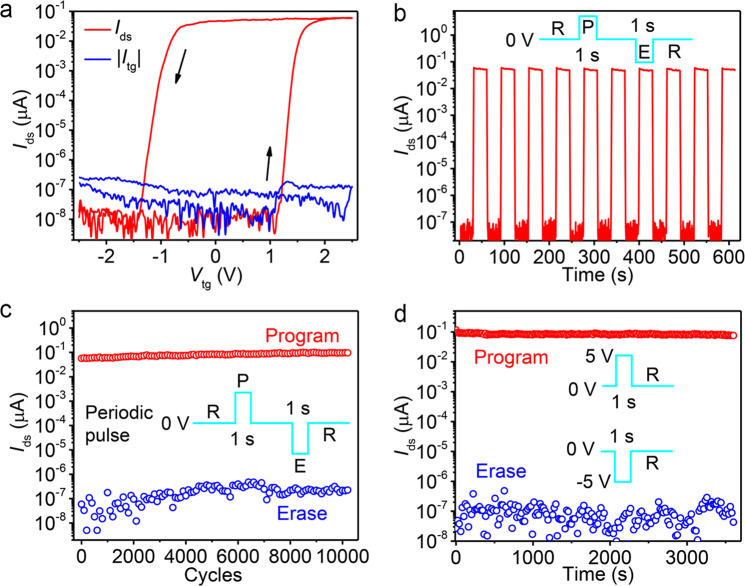
Cycle endurance and time retention characteristics. **a** Top-gate *I*_ds_−*V*_tg_ characteristics (red) and leakage current (black) of a vdW FeFET measured with *V*_ds_ = 0.5 V and internal gate floating. The device has a four-layer MoS_2_ channel and an 86-nm-thick CIPS layer. **b** Dynamic characteristics of the same vdW FeFET in response to periodic top-gate voltage pulses with an amplitude of 3.5 V and width of 1 s. *I*_ds_ was probed with *V*_ds_ = 0.5 V after applying the gate pulse. Inset shows the periodic pulse mode applied to the top gate. **c** Endurance performance through more than 10^4^ P/E cycles. **d** Retention properties of this device at the program and erase states. *I*_ds_ was monitored at *V*_ds_ = 0.5 V and *V*_tg_ = 0 V.

To evaluate the reliability of vdW FeFET for nonvolatile memory applications, we examined the endurance and time retention characteristics in a device with a four-layer MoS_2_ and 86 nm CIPS. A similar counterclockwise hysteresis loop with an MW of ~2.5 V is observed, as shown in Fig. 3a. Two distinct current states with a P/E ratio over 10^7^ at *V*_tg_ = 0 V and *V*_ds_ = 0.5 V are achieved after poling the polarization of CIPS up and down. The top-gate leakage current is below 0.3 pA in the entire gate voltage region, which is at least two orders lower than that of FeFETs with classical ferroelectrics^1,29–31^. The ultra-low subthreshold leakage current (~13 fA) and top-gate leakage current, as the two main contributions of leakage current in vdW FeFETs, promise long-retention and fatigue-free memory performance. We then investigated the dynamic characteristics of the same device by applying periodic voltage pulses with an amplitude of 3.5 V and a width of 1 s to the top-gate electrode. As shown in Fig. 3b, stable switching between the program and erase states with a dynamic P/E ratio greater than 10^6^ is reached at *V*_ds_ = 0.5 V and without any external gate voltage. Figure 3c shows the endurance performance of this vdW FeFET as a function of the number of alternating programs and erase cycles. The P/E switching is reproducible and well-defined with no obvious reduction of the dynamic P/E ratio for over 10^4^ cycles, indicating our memory device is fatigue-free. The stability of the program and erase states, which is crucial for nonvolatile data storage, was further examined by monitoring their time-resolved characteristics. Room-temperature retention characteristics of the same device after a 1 s gate voltage pulse of ±5 V is depicted in Fig. 3d. Two distinct states are maintained with negligible P/E ratio degradation for over one hour.

**Fig. 4:**
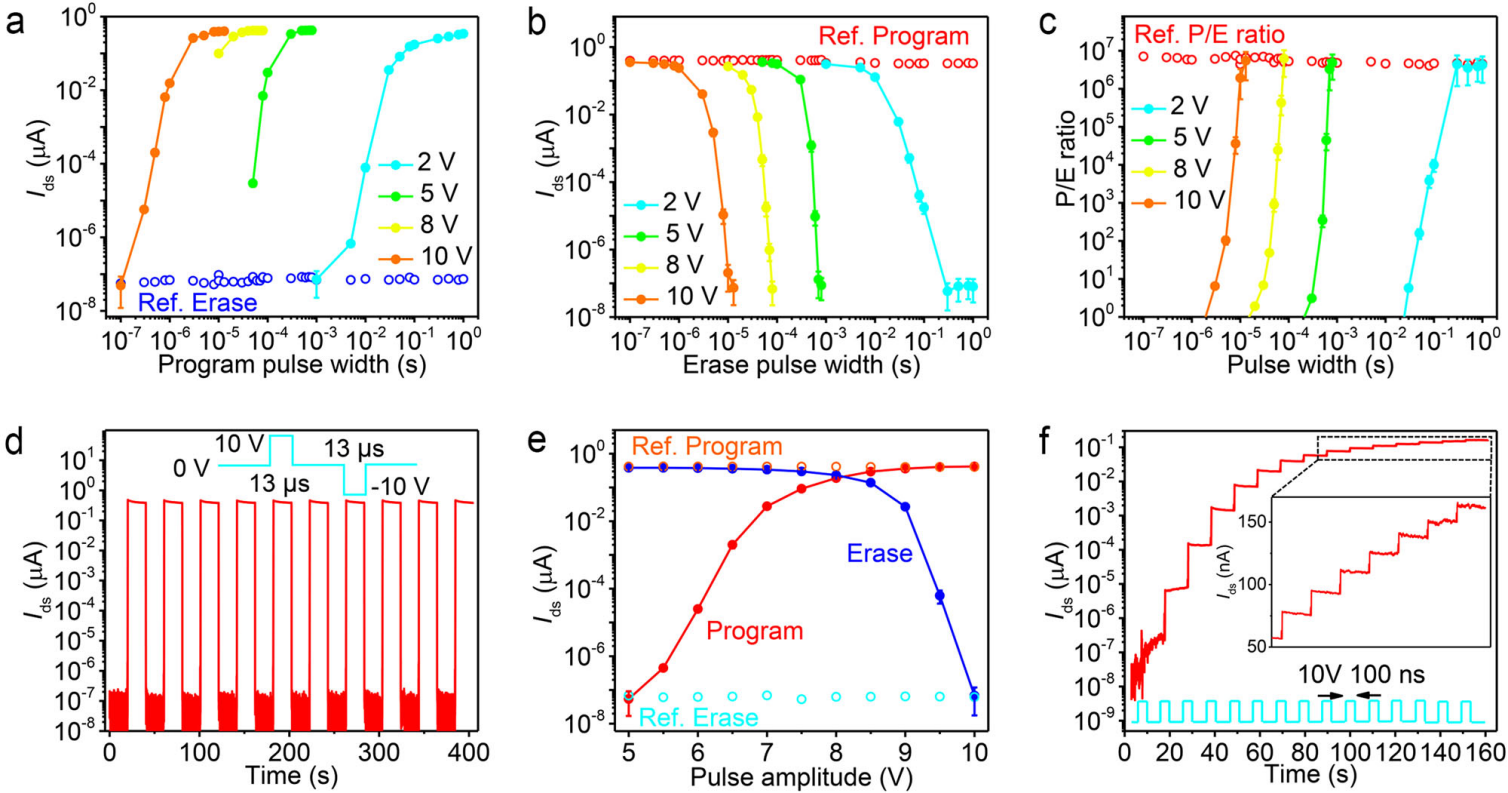
Programing/erasing speed measurements. **a**, **b**, **c** Program **a** and erase **b** state current and the P/E current ratio **c** of a vdW FeFET as a function of *V*_tg_ pulse width for various pulse amplitudes. *I*_ds_ was probed at *V*_ds_ = 0.5 V after applying each pulse. The reference erase (Ref. Erase) and program (Ref. Program) currents were collected after each reset operation but before the writing operation. The device has a six-layer MoS_2_ channel and a 36-nm-thick CIPS layer. **d** Dynamic characteristic of the same device in response to periodic *V*_tg_ pulses with the amplitude of 10 V and width of 13 μs. *I*_ds_ was probed at *V*_ds_ = 0.5 V. **e** *I*_ds_ as a function of the program (red dot) and erase (blue dot) pulse amplitude. The pulse width was fixed at 13 μs. **f** *I*_ds_–time curve of the same vdW FeFET under a 10 V/100 ns pulse train. Dots and bars in **a**, **b**, **c**, and **e** are the mean and standard deviation of *I*_ds_ recorded in 20 s after applying each pulse, respectively.

The programing and erasing speeds of vdW FeFET were further estimated by measuring the gate voltage pulse width dependence of the *I*_ds_ binary state. Figure 4a, b, c illustrate the detailed program and erase characteristics of a vdW FeFET examined by varying pulse amplitude (2 to 10 V) and width (100 ns to 1 s). *I*_ds_–time curves at various top-gate voltage pulses are provided in Supplementary Note 1 and Supplementary Fig. 12–18. Before each program or erase pulse, vdW FeFET was set into the erased or programed state through a reset pulse. As shown in Fig. 4a, when the FeFET is switched from the erase to program state with a 2 V gate voltage pulse, *I*_ds_ increases gradually at first, and then rapidly with increasing pulse width and finally saturates at around 80 ms. Conversely, *I*_ds_ decreases with increasing erase pulse width when the vdW FeFET is switched from the program to erase state, as shown in Fig. 4b. Stable switching between the program and erase states with a P/E ratio of more than 10^6^ is achieved at a 2 V/300 ms gate voltage pulse, as presented in Fig. 4c. No polarization reversal is observed for a 2 V voltage pulse when the pulse width decreases to 1 ms. Writing speed is sensitively dependent on the applied gate voltage amplitude and much shorter time is taken for polarization reversal when the amplitude of *V*_tg_ approaches 10 V. Switching to the program or erase states is completed with a pulse amplitude of 10 V and width of 8 μs or 13 μs, respectively, as shown in Fig. 4a, b. Figure 4d depicts the reproducible P/E characteristics of the same device under an alternating program and erase pulses of ±10 V/13 μs. Note that this vdW FeFET still has a P/E current ratio of more than 2 orders at a writing speed of 5 μs, as shown in Fig. 4c.

The writing voltage was also estimated by measuring the pulse amplitude dependence of *I*_ds_. The program and erase state current as a function of the amplitude of applied *V*_tg_ pulse, with the width fixed, are plotted in Fig. 4e. *I*_ds_ gradually increases with the program pulse amplitude and saturates approximately at 400 nA when the pulse amplitude is greater than 8.5 V. While the erase operation does not saturate before the device is set into an erased state at *V*_tg_ = −10 V. The asymmetric switching characteristics, including the relatively large pulse width and amplitude for erasing compared to programing, are attributed to the partially divided gate voltage by the MoS_2_ channel when a device is switched to the erase state^32^. That varying the program pulse width or amplitude induces various *I*_ds_ offers the possibility of multilevel storage in a vdW FeFET. Figure 4f shows the multilevel *I*_ds_ under 15 gate voltage pulses with an amplitude of 10 V and a width of 100 ns. The stepwise program state is clearly distinct with increasing pulse numbers and 16 levels, corresponding to 4-bit, in 6 orders of drain current with clear gaps. Polarization switching with the current ratio larger than 10^6^ by a single pulse of 100 ns was also observed in a vdW FeFET (Supplementary Fig. 19). Considering the RC delay of the measurement setup, the intrinsic polarization response is expected to be faster than 100 ns. The fast ionic ordering in CIPS simultaneously enhances writing speed and reduces writing voltage in a vdW FeFET. By contrast, it is impossible to decrease the writing speed below several μs with a voltage as low as 10 V in a poly(vinylidene difluoride-trifluoroethylene) (P(VDF-TrFE)) based FeFET^31,33,34^. The operating speed of a vdW FeFET can be further improved by reducing the channel length to accelerate domain wall motion^32^.

**Fig. 5:**
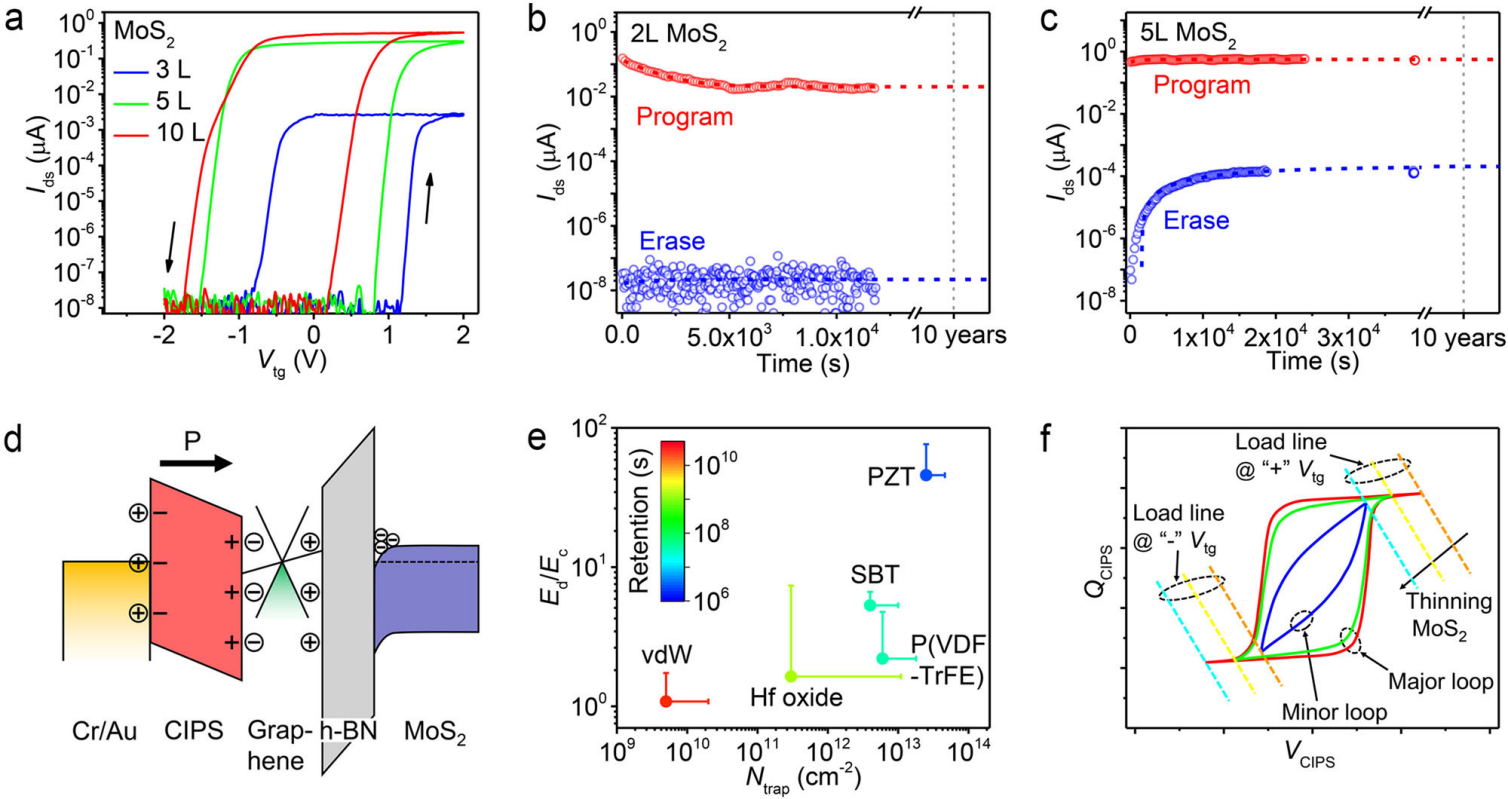
Analysis of memory retention performance of vdW FeFET. **a** Top-gate transfer characteristics of vdW FeFETs with different thickness of MoS_2_. **b**, **c** Program and erase state retention properties of the vdW FeFET with bilayer **b** and five-layer **c** MoS_2_ after voltage pulses of ± 3 V/1 s. *I*_ds_ was monitored at *V*_ds_ = 0.5 V and *V*_tg_ = 0 V. Dashed red and blue lines are the fitting using the exponential decay function. The thickness of CIPS flake is 39 nm (**b**) and 66 nm (**c**). **d** Schematic band diagram and charge distribution of a vdW FeFET during retention time of the program state. **e** Calculated retention time vs. *E*_d_/*E*_c_ ratio and *N*_trap_ for vdW FeFET, P(VDF-TrFE), PZT, SBT, and hafnium oxide based FeFETs. Error bars indicate the range of variation for *E*_d_/*E*_c_ and *N*_trap_. **f** Load-line analysis of layer-dependent retention performance of vdW FeFET. The solid lines are hysteresis loops of MFM capacitor at different statuses. The dashed color lines are load line curves of underlying 2D FET with different thickness of MoS_2_ under the same magnitude of *V*_tg_.

The memory characteristics of a vdW FeFET were then comprehensively investigated by varying the thickness of the MoS_2_ layer. As shown in Fig. 5a, increasing the thickness of MoS_2_ enhances the conductance and P/E ratio. However, the threshold voltage (*V*_th_) of the FeFET is reduced due to the increased work function for a thicker MoS_2_. Further reducing the *V*_th_ shifts MW completely to the negative *V*_tg_ region and deteriorates the memory performance. To achieve a sufficient P/E ratio and long retention time, MoS_2_ with a layer number less than 10 was selected as the FeFET channel in this work. The retention properties of FeFETs with bilayer and five-layer MoS_2_ are given in Fig. 5b, c, respectively. The current ratio of P/E retention is above 10^6^ after more than 3 h for the bilayer MoS_2_ FeFET and 4 × 10^3^ after more than 10 h for the five-layer MoS_2_ FeFET. As indicated by the dashed gray lines in Fig. 5b, c, the program and erase states can be clearly distinguished up to an extrapolation of 10 years retention for both devices, demonstrating the great potential of vdW FeFETs for nonvolatile memory applications.

## Discussion

Two major mechanisms have been proposed to account for the retention loss in FeFET:^11,20,35^ 1) a depolarization field arises from incomplete charge compensation, which induces the fast retention loss at the early stage of time evolution, and 2) carrier charge trapping, which is responsible for the retention loss in the longer time regime. The rate of early-stage retention loss is determined by the ratio of the depolarization field (*E*_d_) to coercive field (*E*_c_) of FeFET^21^, where *E*_*d*_ = *P*[*ε*_*CIPS*_ (*C*_*IS*_/*C*_*CIPS*_ + 1)]^−1^ (Supplementary Note 2 and Supplementary Fig. 20) and *P*, *ε*_CIPS_, and *C*_CIPS_ are the ferroelectric polarization, dielectric constant, and capacitance of CIPS, and *C*_IS_ is the series combination of the capacitance of h-BN and MoS_2_ channel. The retention loss induced by charge trapping is proportional to the trap density (*N*_trap_)^35^. From these discussions, vdW FeFET affords the full advantages from material to structure in preventing retention loss induced by both mechanisms. The nearly squared *P*−*E* loop of CIPS with a large coercive field and moderate remnant polarization leads to a weak depolarization effect. Note that the remnant polarization of CIPS (in the range of 2.5−4.0 μC/cm^2^)^15,36,37^ matches well with the charges required for the underlying 2D FET, the maximum value of which is calculated to be 3.99 μC/cm^2^. This enables a vdW FeFET operating with a major ferroelectric hysteresis loop and makes it invulnerable to the internal depolarization field^38^. As illustrated by the band diagrams of Fig. 5d, the employment of MFMIS structure moves compensating charges close to the surface of the ferroelectric, leaving an eliminated residual depolarization field. Gate leakage followed by trapping can also be suppressed through this vdW MFMIS structure due to the high barrier of h-BN and ideal defect-free surfaces. Figure 5e illustrates the comparison of calculated retention performance for vdW FeFET, PZT, SBT, P(VDF-TrFE), and hafnium oxide based FeFETs, where one can see vdW FeFET exhibits much lower *E*_d_/*E*_c_ and *N*_trap_ (as low as 5.2 × 10^9^ cm^−2^)^39,40^ compared with the other FeFETs. Therefore, vdW FeFET is much more likely to achieve a memory retention time of more than 10 years. The details for the estimation of retention performance of FeFETs are provided in Supplementary Note 3 and Supplementary Table 1. The asymmetric retention property between the program and erase states in Fig. 5b, c is ascribed to the difference in operating point. As schemed in Fig. 5f, the operating point for a FeFET, with a thinner (thicker) MoS_2_ at the programing (erasing) phase, sets up on the minor hysteresis loop, making the program (erase) state relatively vulnerable to the depolarization field. While the major loop is utilized for FeFET with a thinner (thicker) MoS_2_ during erasing (programing). This is also supported by the observed symmetric retention characteristic of the vdW FeFET with a four-layer MoS_2_ (shown in Fig. 3), where the MW is located with centering around *V*_tg_ = 0 V and the major loop is employed under the same magnitude of gate voltage for both programing and erasing operations. The finally saturated current in Fig. 5b, c indicate much less trapping-related retention loss, which benefits from the trap-free vdW interfaces.

Table 1 summarizes the main features of the vdW FeFET benchmarked against the reported FeFET memory devices that employ P(VDF-TrFE), PZT and ferroelectric hafnium oxide as the gate dielectric. Among these devices, the vdW FeFET exhibits comparable memory performance with the hafnium oxide based FeFETs and better performance in operation speed than the P(VDF-TrFE) based FeFETs.

**Table 1:** Comparison of this work and previous works that employed ferroelectric P(VDF-TrFE), PZT and hafnium oxide as the gate dielectric of FeFET.

Ferroelectric	Channel	P/E ratio	P/E speed	Endurance	Retention
P(VDF-TrFE)^31^	MoSe_2_	> 10^5^	50 μs/2 ms	10^4^	> 2 × 10^3^ s
P(VDF-TrFE)^33^	MEH-PPV	> 10^4^	500 μs/50 μs	10^3^	2 × 10^5^ s
PZT^41^	IGZO	10^6^	3 s	–	10^3^ s
PZT^42^	ZnO	10^5^	150 ms	–	10 years
Si:HfO_2_^43^	Si	≈10^3^	10 μs	2 × 10^4^	1 h
Si:HfO_2_^44^	Si	> 100	1–10 μs	10^5^	72 h
HZO^45^	Si	10^6^	100 μs	10^7^	10 years
HZO^46^	Si	10^4^	0.5 μs	10^6^	10 years
CIPS (this work)	MoS_2_	> 10^7^	5 μs	> 10^4^	10 years

In summary, nonvolatile memory FeFETs have been demonstrated with the MFMIS structure entirely built from all vdW material components. The vdW FeFET features sub-20 mV dec^−1^ operation, a MW larger than 3.8 V, and a current ratio greater than 10^7^ at zero gate voltage. Deterioration is negligible even after more than 10^4^ cycles of P/E endurance. A memory retention time of more than 10 h extrapolated to 10 years is achieved at room temperature. Sub-5 μs writing speed is demonstrated, and even a single pulse with a width of 100 ns pulses is enough for polarization reversal. Moreover, the gradual polarization switching behavior and high P/E current ratio of vdW FeFETs allow 16 distinct storage states per cell. We suggest that long memory retention, high endurance, and fast writing speed make the present device architecture a suitable candidate for nonvolatile memory applications. Also, our work reveals that vdW engineering is a practical solution to the ferroelectric memory retention problem.

## Methods

### Heterostructure preparation and device fabrication

Single crystals of CIPS and MoS_2_ were synthesized by solid-state reaction, and h-BN was produced by the atmospheric pressure metal flux method^19^. All the vdW flakes, including MoS_2_, h-BN, graphene, and CIPS, were achieved by mechanical exfoliation from bulk crystals. The heterostructures were produced with a dry transfer technique as reported. A few-layer MoS_2_ flake was first exfoliated onto a Si substrate covered with a 300-nm-thick SiO_2_ layer. BN, graphene, and CIPS flakes were exfoliated onto transparent poly-dimethylsiloxane (PDMS) films. A PDMS film with an h-BN flake was then stamped onto an MoS_2_ flake with the aid of a micromanipulator under an optical microscope. The h-BN flake was released and transferred onto the MoS_2_ flake after the PDMS film was lifted off. Next, graphene and the CIPS flake were transferred onto the h-BN with the same method. Finally, Cr/Au (5 nm/80 nm) electrodes were defined on the fabricated heterostructure using the standard e-beam lithography process followed by the metal thermal evaporation and lift-off process. To fabricate the parallel-plate capacitors, CIPS flakes were exfoliated onto a Si/SiO_2_ wafer covered with a Cr (5 nm)/Au (30 nm) metal layer and Cr (5 nm)/Au (80 nm) was then deposited on the surface of CIPS flakes as the top electrodes.

### Characterizations

Atomic force microscopy (AFM, Asylum Research Cypher S) in a tapping mode was used to characterize the morphology of the heterostructures and devices. The layer number of the MoS_2_ was identified by AFM or micro-Raman spectroscopy (Witec) with a 532 nm laser. Out-of-plane PFM measurements were carried out on the Cypher S AFM in the DART mode. Off-field PFM hysteresis loops were measured by recording the piezoresponse amplitude and phase signals after the individual DC pulse was turned off. *P*−*E* hysteresis loop measurements were carried out using a Radiant ferroelectric tester with an applied voltage of a triangular waveform at 1 kHz. Static transport properties were measured with an Agilent B1500A Semiconductor Device Parameter Analyzer in a vacuum chamber of 10^−2^ torr. The dynamic and writing speed tests were performed with the top-gate electrode connected to a RIGOL DG1032Z signal generator and source and drain connected to the Agilent B1500A.

## Supplementary information

Supplementary Information

## Data Availability

The data that support the findings of this study are available from the corresponding author upon reasonable request.
