# Author Correction: Van der Waals engineering of ferroelectric heterostructures for long-retention memory

**DOI:** 10.1038/s41467-021-23392-6

**Published:** 2021-05-10

**Authors:** Xiaowei Wang, Chao Zhu, Ya Deng, Ruihuan Duan, Jieqiong Chen, Qingsheng Zeng, Jiadong Zhou, Qundong Fu, Lu You, Song Liu, James H. Edgar, Peng Yu, Zheng Liu

**Affiliations:** 1 School of Materials Science and Engineering, Nanyang Technological University, Singapore, Singapore; 2 Jiangsu Key Laboratory of Thin Films, School of Physical Science and Technology, Soochow University, Suzhou, China; 3 Tim Taylor Department of Chemical Engineering, Durland Hall, Kansas State University, Manhattan, KS USA; 4 School of Materials Science and Engineering, State Key Laboratory of Optoelectronic Materials and Technologies, Sun Yatsen University, Guangzhou, China; 5 CINTRA CNRS/NTU/THALES, UMI 3288, Research Techno Plaza, Singapore, Singapore; 6 Centre for Micro-/Nanoelectronics (NOVITAS), School of Electrical and Electronic Engineering, Nanyang Technological University, Singapore, Singapore

**Keywords:** Information storage, Two-dimensional materials, Electronic devices

Correction to: *Nature Communications* 10.1038/s41467-021-21320-2, published online 17 February 2021.

The original version of this Article contained an error in the Acknowledgements:

NRF-CRP22-2019-0060 should have read NRF-CRP22-2019-0007.

This has now been corrected in both the PDF and HTML versions of the Article.

