# Supplementary material for: Van der Waals engineering of ferroelectric heterostructures for long-retention memory
Source: Nat Commun. 2021 Feb 17;12:1109. doi: 10.1038/s41467-021-21320-2 (PMC7889872; doi:10.1038/s41467-021-21320-2)
Supplement: Supplementary file 1 — Supplementary Information [file 41467_2021_21320_MOESM1_ESM.pdf]

Van der Waals engineering of ferroelectric heterostructures for long-retention memory

Xiaowei Wang[1], Chao Zhu[1], Ya Deng[1], Ruihuan Duan[1], Jieqiong Chen[1], Qingsheng Zeng[1], Jiadong Zhou[1], Qundong Fu[1], Lu You[2], Song Liu[3], James H. Edgar[3], Peng Yu[4], Zheng Liu[1,5,6*]

[1]School of Materials Science and Engineering, Nanyang Technological University, Singapore 639798, Singapore.

[2]Jiangsu Key Laboratory of Thin Films, School of Physical Science and Technology, Soochow University, Suzhou 215006, China.

[3]Tim Taylor Department of Chemical Engineering, Durland Hall, Kansas State University, Manhattan, KS 66506, USA.

[4]School of Materials Science and Engineering, State Key Laboratory of Optoelectronic Materials and Technologies, Sun Yat-sen University, Guangzhou 510275, China.

[5]CINTRA CNRS/NTU/THALES, UMI 3288, Research Techno Plaza, Singapore 637553, Singapore.

[6]Centre for Micro-/Nano-electronics (NOVITAS), School of Electrical and Electronic Engineering, Nanyang Technological University, Singapore.

*e-mail: z.liu@ntu.edu.sg

Contents

Supplementary Figures......3

Supplementary Figure 1. Fabrication process of vdW heterostructure based FeFETs......3

Supplementary Figure 2. AFM characterizations of a vdW heterostructure......4

Supplementary Figure 3. MoS_2 layer number determination......5

Supplementary Figure 4. Domain area of CIPS......6

Supplementary Figure 5. Raw data of Figure 1e and f......7

Supplementary Figure 6. Ferroelectric polarization switching in CIPS......8

Supplementary Figure 7. Optical image of a CIPS parallel-plate capacitor......9

Supplementary Figure 8. Memory performance of vdW FeFETs with different thickness of CIPS......10

Supplementary Figure 9. Memory performance of the MFS FeFET......11

Supplementary Figure 10. Sub-60 mV dec^{-1} switch in vdW FeFETs......12

Supplementary Figure 11. The I_{ds}–V_{tg} characteristics of the same device as in Figure 2f......13

Supplementary Figure 12. The V_{tg} pulse sequence used to measure the writing speed......14

Supplementary Figure 13. Writing speed test at V_{tg} = 2 V. T......15

Supplementary Figure 14. Writing speed test at V_{tg} = 5 V. T......16

Supplementary Figure 15. Writing speed test at V_{tg} = 8 V......17

Supplementary Figure 16. Writing speed test at V_{tg} = 10 V. T......18

Supplementary Figure 17. V_{tg} pulse sequence used to measure P/E voltage......19

Supplementary Figure 18. The P/E voltage test......20

Supplementary Figure 19. Polarization switching with a 100 ns single pulse......21

Supplementary Figure 20. Equivalent capacitor network of a vdW FeFET......22

Supplementary Tables......23

Supplementary Table 1. Parameters for the calculation of FeFET retention time......23

Supplementary Notes......24

Supplementary Note 1: Writing speed and voltage test......24

Supplementary Note 2: Depolarization field in a vdW FeFET......25

Supplementary Note 3: Calculation of the retention time of a FeFET......26

Reference......27

Supplementary Figures

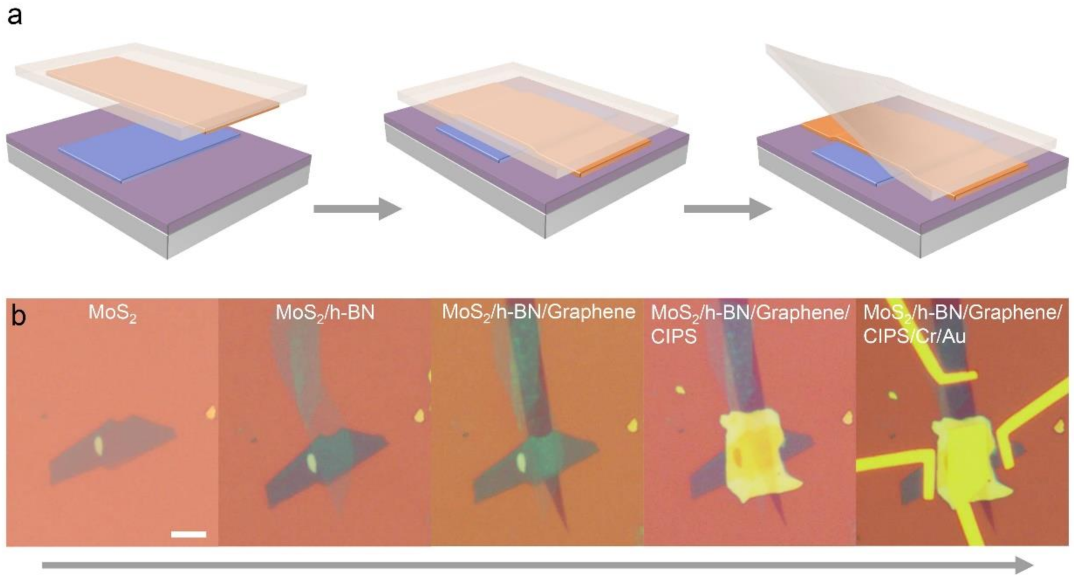

Supplementary Figure 1. Fabrication process of vdW heterostructure based FeFETs. a, Schematic of the dry transfer process for creating vdW heterostructure. A 2D flake was exfoliated onto a transparent poly-dimethylsiloxane (PDMS) film and then stamped onto another flake on a SiO_2/Si substrate with the aid of a micromanipulator under an optical microscope. After a slight press and lift-off of the PDMS, the flake on the PDMS was released, and the vdW heterostructure was achieved. **b**, Optical microscope images of a vdW FeFET at different process stages. Scale bars, 5 μm.

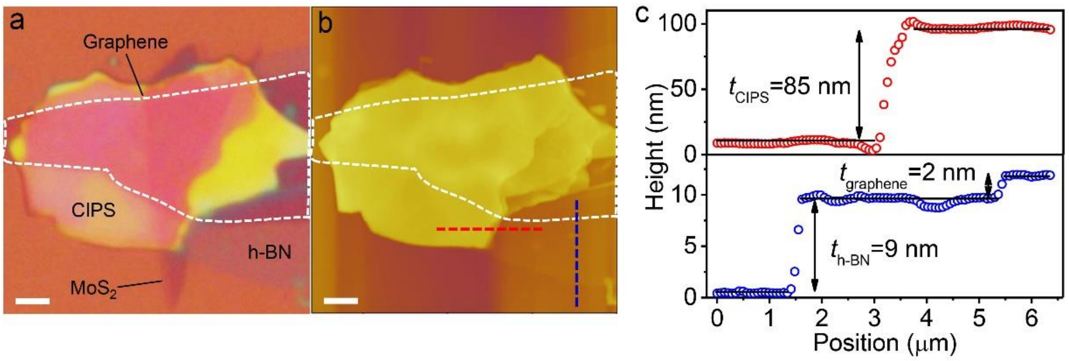

Supplementary Figure 2. AFM characterizations of a vdW heterostructure. Optical microscope (**a**) and AFM topography (**b**) image of the MoS_2/h-BN/graphene/CIPS heterostructure shown in Figure 1c and d, Scale bar, 2 μm. **c**, Height profiles along the dashed line as indicated in **b**.

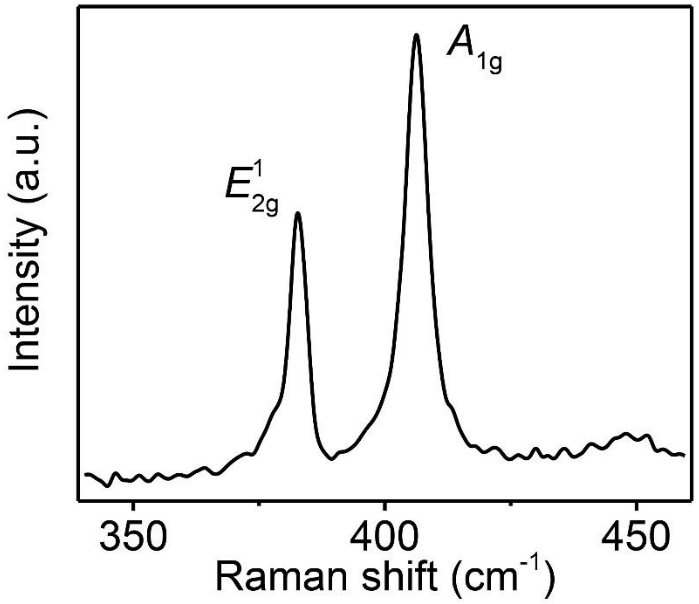

Supplementary Figure 3. MoS_2 layer number determination. The layer number (*n*) of MoS_2 is determined through Raman spectroscopy when $n \leq 6$. There are two characteristic Raman modes for MoS_2, the in-plane (E^1_{2g}) and the out-of-plane (A_{1g}) vibrational modes with a frequency difference of $\Delta\omega$ = 23.5 cm^{-1} indicating a trilayer MoS_2[1]. AFM was used to determine the layer number of MoS_2 with $n > 6$.

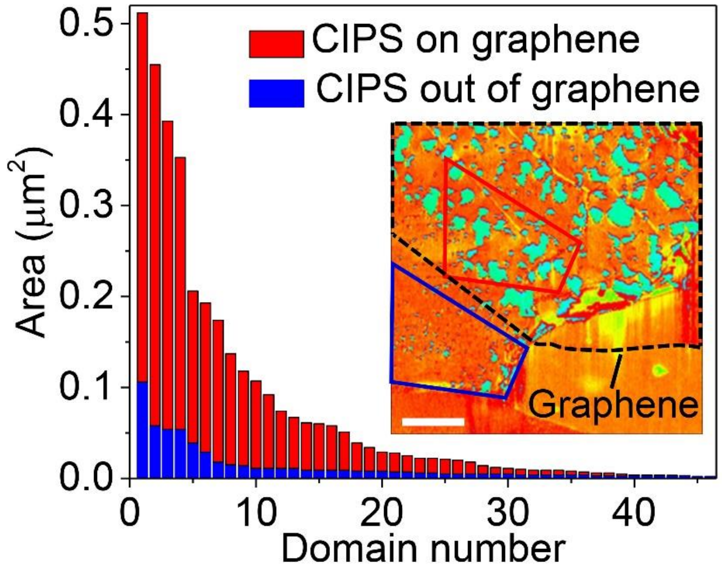

Supplementary Figure 4. Domain area of CIPS. Measured domain area of CIPS on (red) and out of (blue) graphene. The inset shows the same PFM phase image as in Figure 1d of the main text. Scale bar, 2 μm. 46 domains were collected from the red and blue lines enclosed region of PFM phase image, which have the same area. The averaged domain area of CIPS on graphene (0.063 μm^2) is ~5 times of that out of graphene (0.013 μm^2).

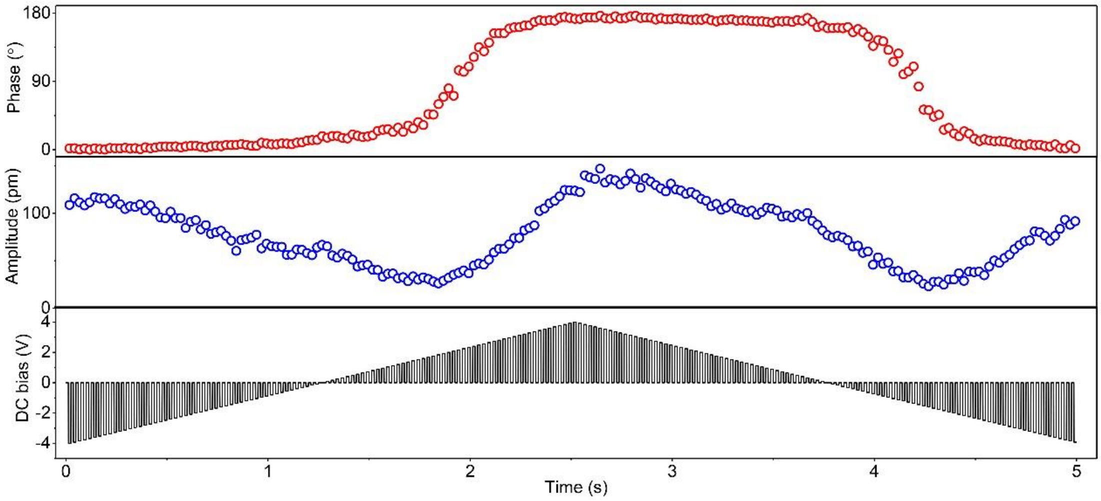

Supplementary Figure 5. Raw data of Figure 1e and f. DC bias, PFM amplitude, PFM phase of CIPS sample shown in Figure 1e and f as a function of time.

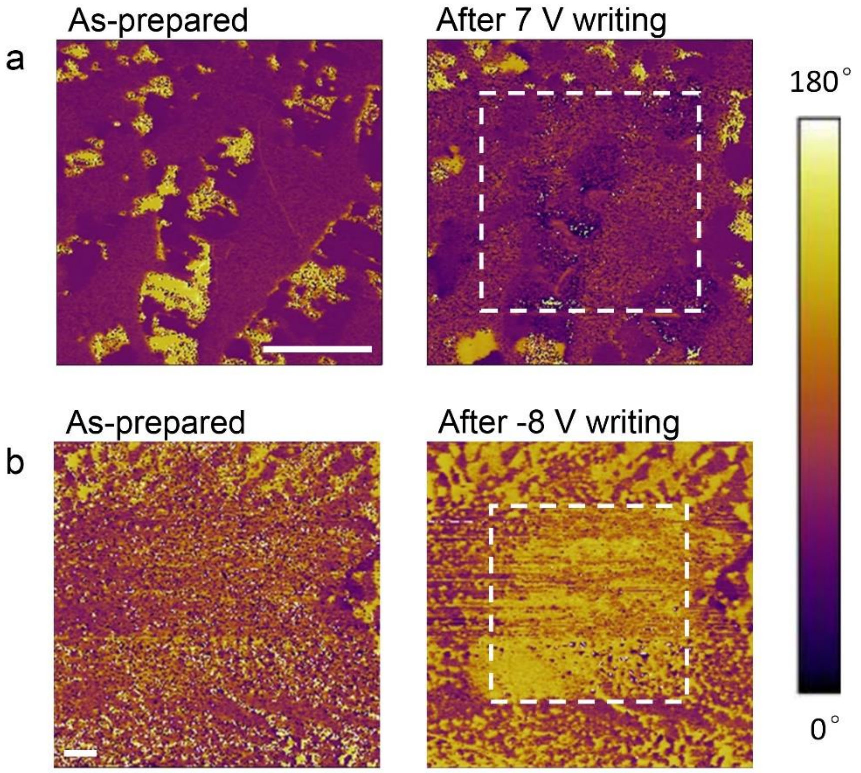

Supplementary Figure 6. Ferroelectric polarization switching in CIPS. The PFM phase images of a CIPS flake before (left) and after (right) writing a square pattern at the region enclosed by dashed lines with an (**a**) 7 V and (**b**) -8 V DC bias. Scale bar, 1 μm. Clear reversal of phase contrast confirms the switching of polarization in CIPS.

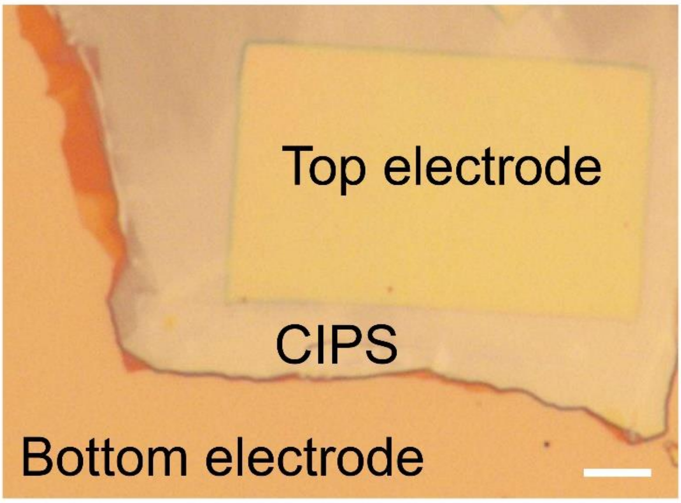

Supplementary Figure 7. Optical image of a CIPS parallel-plate capacitor. Scale bar, 5 μm.

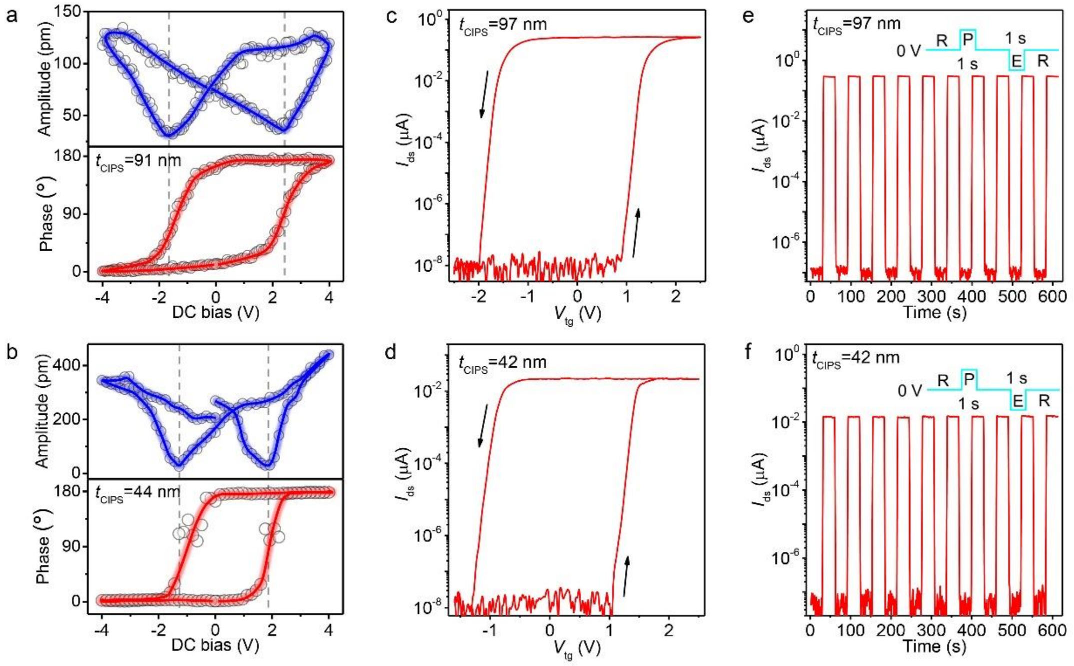

Supplementary Figure 8. **Memory performance of vdW FeFETs with different thickness of CIPS.** Off-field PFM amplitude (blue) and phase (red) hysteresis loops on a (**a**) 91 nm and (**b**) 44 nm-thick CIPS flake. Top-gate I_{ds}–V_{tg} characteristics of the vdW FeFET with (**c**) 97 nm CIPS and (**d**) 42 nm CIPS. V_{ds} = 0.5 V. Thinning CIPS results in a decreased memory window of vdW FeFET due to the reduced coercive voltage and the improved capacitance matching condition. To achieve a large memory window, CIPS with the thickness larger than 30 nm was selected as the FeFET ferroelectric dielectric in this work. Dynamic characteristics of the same vdW FeFET with (**e**) 97 nm CIPS and (**f**) 42 nm CIPS in response to periodic top-gate voltage pulses. I_{ds} was probed with V_{ds} = 0.5 V after applying the gate pulse. Inset shows the periodic pulse mode applied to the top gate. Stable switching between the program and erase states with a dynamic P/E ratio greater than 10^5 are reached for both devices at V_{ds} = 0.5 V and without any external gate voltage.

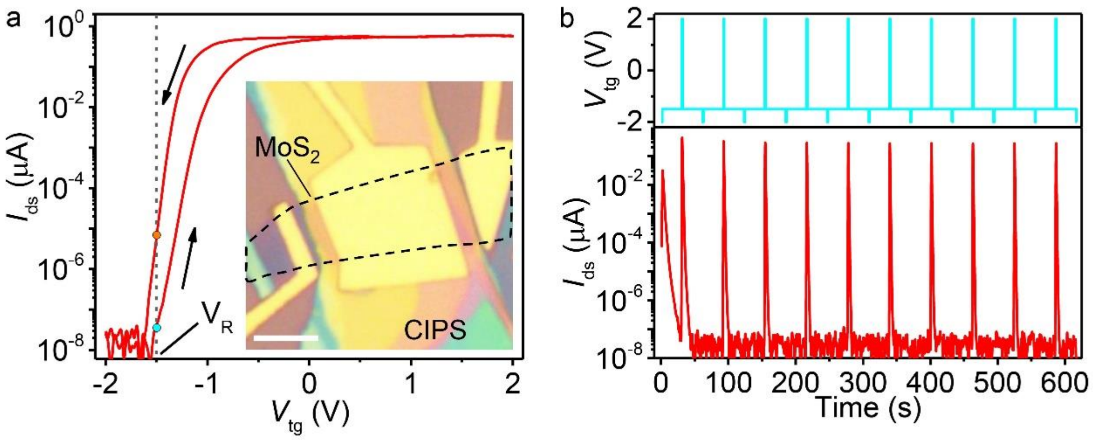

Supplementary Figure 9. Memory performance of the MFS FeFET. (**a**) Top-gate I_{ds}–V_{tg} characteristics of the MFS FeFET, V_{ds} = 0.5 V. Inset: Optical image of the corresponding FeFET. Scale bar, 5 μm. The I_{ds}–V_{tg} curve of MFS FeFET exhibits an anticlockwise hysteresis loop with a very small memory window (< 0.2 V) at the negative gate voltage region. (**b**) Dynamic characteristics (bottom) of the same device in response to periodic top-gate voltage pulses (top). I_{ds} was probed with V_{ds} = 0.5 V and V_{tg} = -1.5 V after applying the gate pulse and no memory effect is observed.

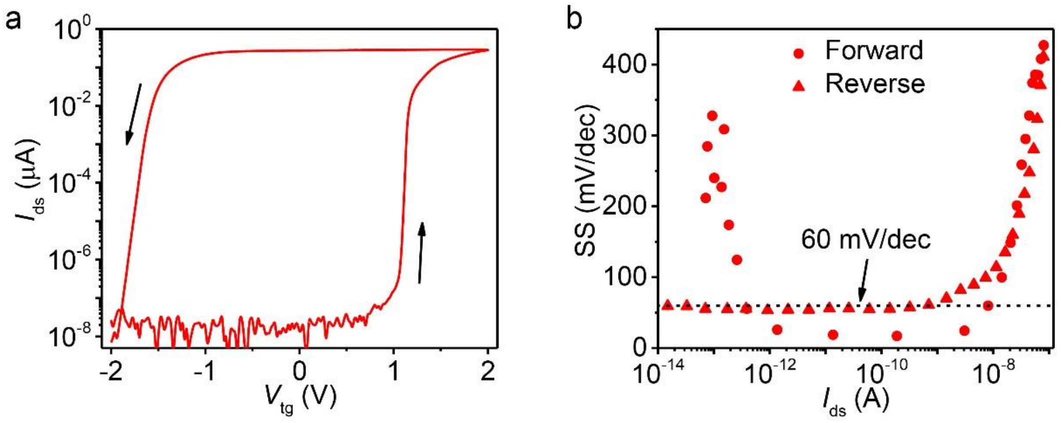

Supplementary Figure 10. Sub-60 mV dec^{-1} switch in vdW FeFETs. a, The I_{ds}–V_{tg} characteristics of a vdW FeFET with a 66 nm CIPS and five-layer MoS_2. **b**, The SS versus I_{ds} characteristics extracted from the I_{ds}–V_{bg} curves in **a**. The vdW FeFET exhibits sub-60 mV dec^{-1} switch for both the forward and reverse sweep, with a minimum SS of 17 mV dec^{-1} for the forward sweep and 53 mV dec^{-1} for the reverse sweep.

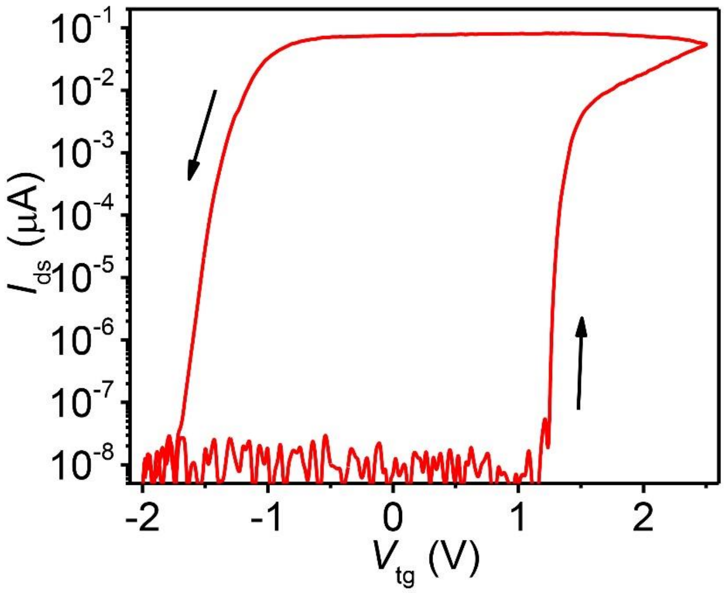

Supplementary Figure 11. The I_{ds}−V_{tg} characteristics of the same device as in Figure 2f. The device has a bilayer MoS_2 channel and a 39-nm-thick CIPS layer. The device shows an MW of ~2.9 V and P/E current ratio of >10^7. A coercive voltage of ~1.2 V is determined from the reverse sweep of the I_{ds}−V_{tg} curve.

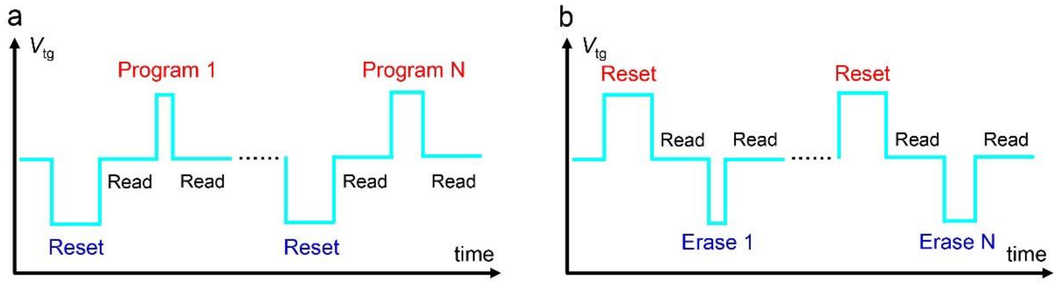

Supplementary Figure 12. The V_{tg} pulse sequence used to measure the writing speed. **a**, V_{tg} waveform scheme for varying program pulse width. **b**, V_{tg} waveform scheme for varying erase pulse width.

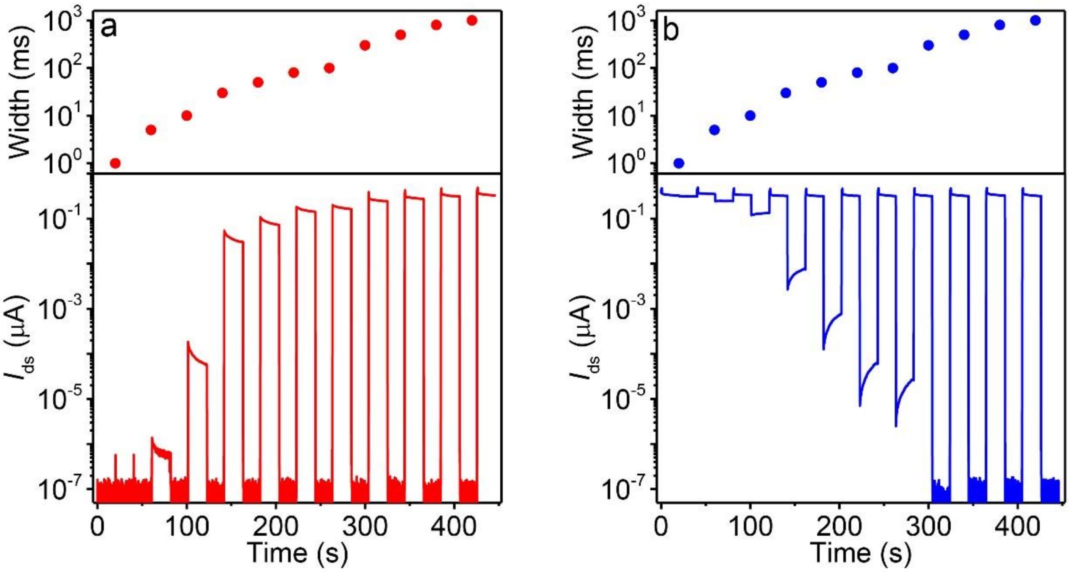

Supplementary Figure 13. Writing speed test at V_{tg} = 2 V. Time-dependent I_{ds} at different program (**a**) and erase (**b**) voltage pulse widths with a pulse amplitude of 2 V. The upper plot corresponds to the pulse width. I_{ds} was recorded at V_{ds} = 0.5 V for 20 s after each program or erase pulse. Prior to each program or erase pulse, the FeFET was reset into an erase or program state by applying an initialization pulse of −2 V/1 s or +2 V/1 s, respectively.

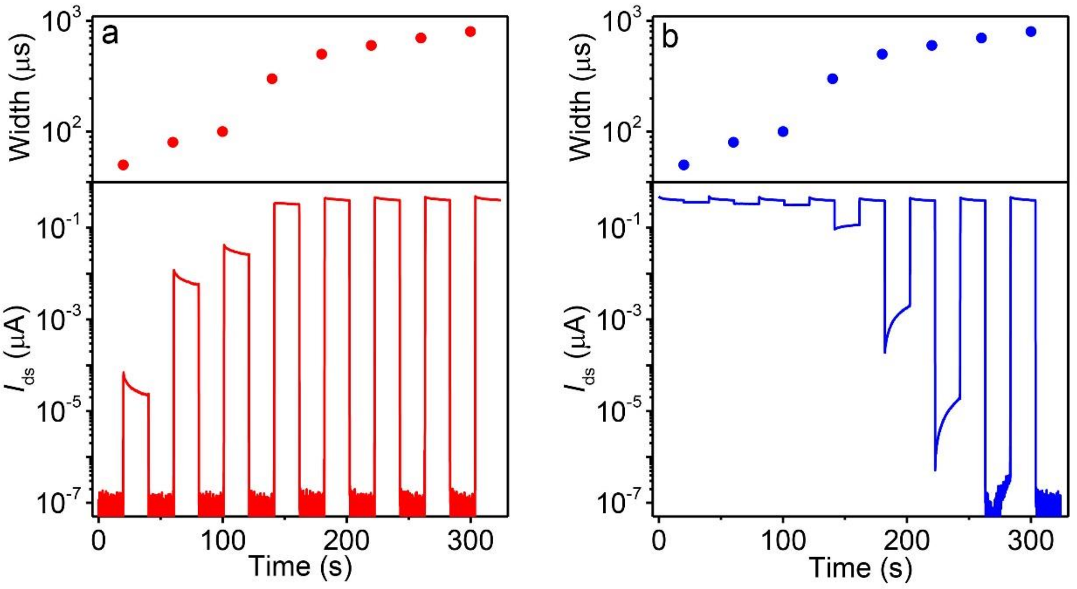

Supplementary Figure 14. Writing speed test at V_{tg} = 5 V. Time-dependent I_{ds} at different program (**a**) and erase (**b**) voltage pulse widths with a pulse amplitude of 5 V. The upper plot corresponds to the pulse width. I_{ds} was recorded at V_{ds} = 0.5 V for 20 s after each program or erase pulse. Prior to each program or erase pulse, the FeFET is reset into an erase or program state by applying an initialization pulse of −5 V/800 μs or +5 V/800 μs, respectively.

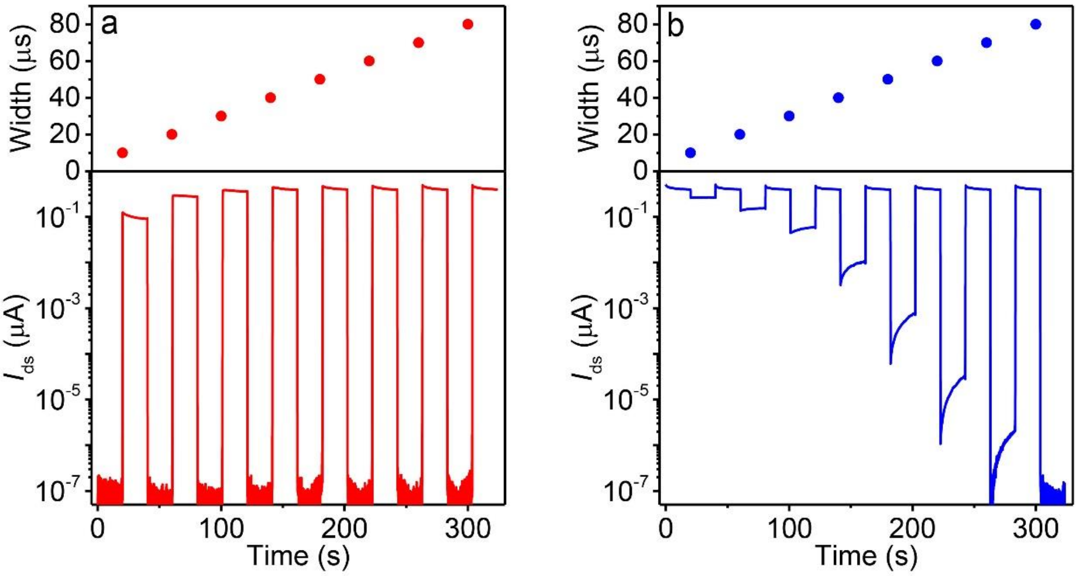

Supplementary Figure 15. Writing speed test at V_{tg} = 8 V. Time-dependent I_{ds} at different program (**a**) and erase (**b**) voltage pulse widths with a pulse amplitude of 8 V. The upper plot corresponds to the pulse width. I_{ds} was recorded at V_{ds} = 0.5 V for 20 s after each program or erase pulse. Prior to each program or erase pulse, the FeFET is reset into an erase or program state by applying an initialization pulse of −8 V/80 μs or +8 V/80 μs, respectively.

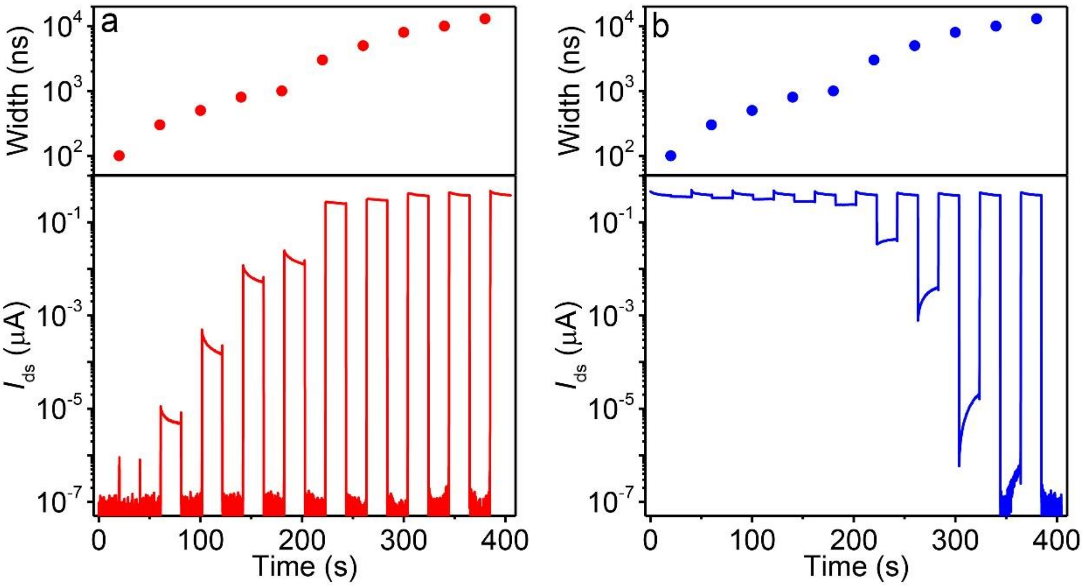

Supplementary Figure 16. Writing speed test at V_{tg} = 10 V. Time-dependent I_{ds} at different program (**a**) and erase (**b**) voltage pulse widths with a pulse amplitude of 10 V. The upper plot corresponds to the pulse width. I_{ds} was recorded at V_{ds} = 0.5 V for 20 s after each program or erase pulse. Prior to each program or erase pulse, the FeFET is reset into an erase or program state by applying an initialization pulse of −10 V/13 μs or +10 V/13 μs, respectively.

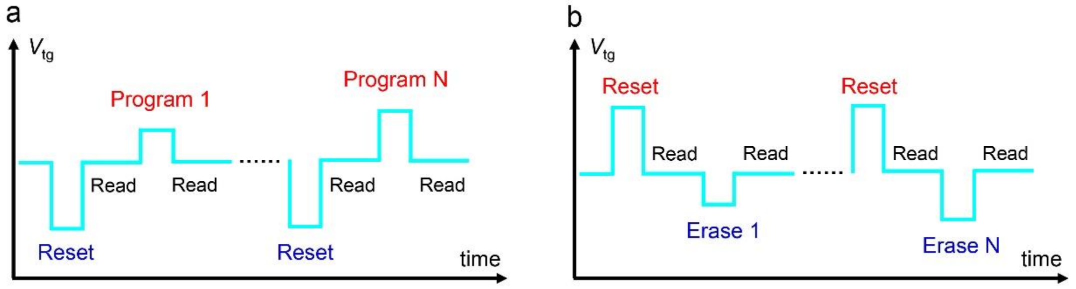

Supplementary Figure 17. V_{tg} pulse sequence used to measure P/E voltage. a, V_{tg} waveform scheme for varying program pulse amplitudes with the pulse width fixed. **b**, V_{tg} waveform scheme for varying erase pulse amplitudes with the pulse width fixed.

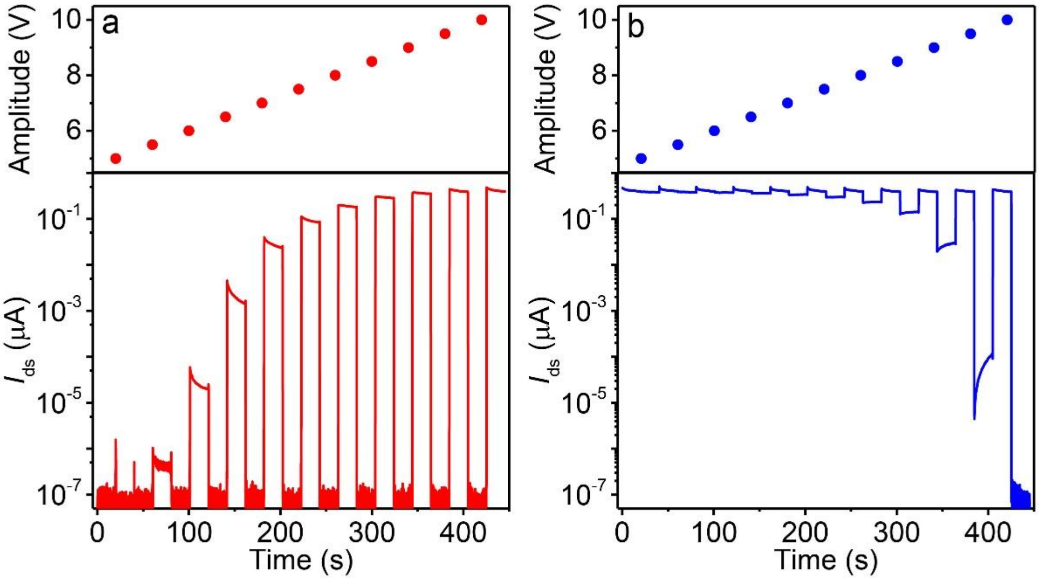

Supplementary Figure 18. The P/E voltage test. Time-dependent I_{ds} at different program (**a**) and erase (**b**) voltage pulse amplitudes with a pulse width of 13 μs. The upper plot corresponds to the pulse amplitude. I_{ds} was recorded at V_{ds} = 0.5 V for 20 s after each program or erase pulse. Prior to each program or erase pulse, the FeFET was reset into an erase or program state by applying an initialization pulse of −10 V/13 μs or +10 V/13 μs, respectively.

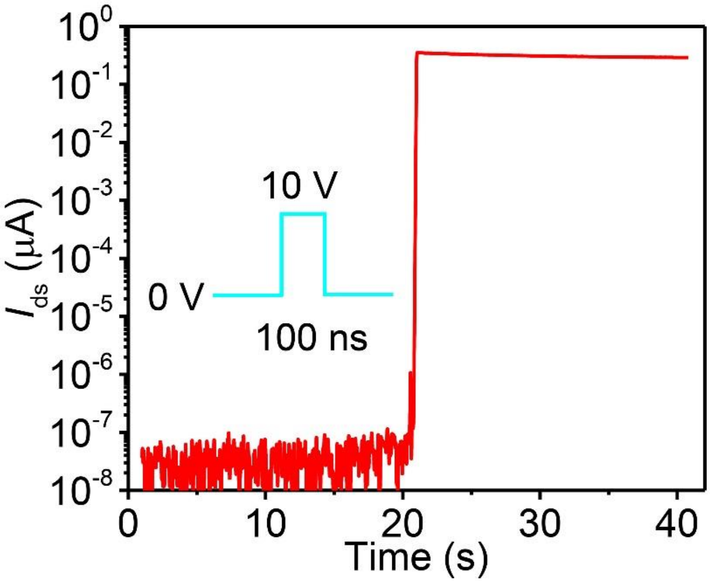

Supplementary Figure 19. Polarization switching with a 100 ns single pulse. The time response of a vdW FeFET with a single voltage pulse of 10 V/100 ns. A clear I_{ds} transition from the off to on state with the current ration over 10^6 was observed, indicating sub-100 ns response time of the vdW FeFET.

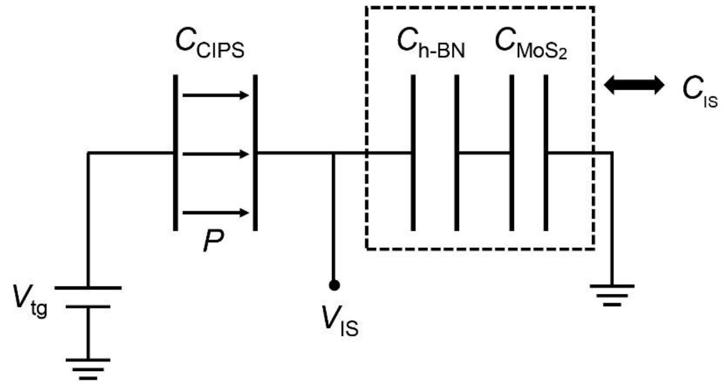

Supplementary Figure 20. Equivalent capacitor network of a vdW FeFET. The gate stack of a vdW FeFET is modeled by a ferroelectric capacitance (C_{CIPS}) in series with the dielectric capacitance ($C_{h\text{-}BN}$) and semiconductor capacitance (C_{MoS2}). C_{IS} is used to represent the series combination of $C_{h\text{-}BN}$ and C_{MoS2}. The applying of a gate voltage V_{tg} induces a polarization P in CIPS and a voltage drop V_{IS} across C_{IS}.

Supplementary Tables

Ferroelectric	P_r (μC/cm^2)	ε	E_c (kV/cm)	N_{trap} (cm^{-2})
P(VDF-TrFE)[2-5]	6–13	15	570	6×10^{12}–1.8×10^{13}
SBT[2,6]	8–10	150	90	4×10^{12}–1×10^{13}
PZT[2,6]	30–50	200	31	2.5×10^{13}–4.6×10^{13}
HfO_2[6-9]	10–45	30	1000	3×10^{11}–1.1×10^{13}
CIPS[10-15]	2.5–4	40	330	5×10^{9}–2×10^{10}

Supplementary Table 1. Parameters for the calculation of FeFET retention time.

Supplementary Notes

Supplementary Note 1: Writing speed and voltage test

The writing speed and voltage of a vdW FeFET were estimated by examining the switching transition characteristics at various gate pulses. A signal generator connected to the top-gate electrode was used to apply the voltage pulse, and a semiconductor parameter analyzer connected to the source and drain electrodes was used to probe the time response of the drain current. **Supplementary Figure 12** shows the top-gate voltage pulse sequences applied during the program and erase speed measurements. Prior to each program pulse, the device was set into an erase state with a negative initialization pulse. The program pulse width was varied at each program voltage. I_{ds} was recorded at V_{ds} = 0.5 V for 20 s with a sampling interval of 40 ms after applying each reset and program pulse. Conversely, I_{ds} was recorded after applying the erase and positive reset pulses for determining the erase speed. **Supplementary Figures 13** to **16** show the I_{ds}-time characteristics at the various program and erase pulses of the same device shown in Figure 4. I_{ds} measured after each program (erase) pulse was then plotted as a function of the pulse width with I_{ds} collected after each reset operation but prior to the program (erase) operation as the reference, which is shown in Figure 4a (b).

The writing voltage of the vdW FeFET was also examined by varying the pulse amplitude at a fixed pulse width of 13 μs. **Supplementary Figure 17** shows the gate voltage pulse sequences applied during the writing voltage measurements. I_{ds} was probed at V_{ds} = 0.5 V for 20 s with a sampling interval of 40 ms after applying each reset and writing pulse. I_{ds} as a function of the pulse amplitude, as shown in Figure 4e,

for determining the writing voltage was then extracted from **Supplementary Figure 18**.

Supplementary Note 2: Depolarization field in a vdW FeFET

The top-gate stack of the vdW FeFET is described with a ferroelectric capacitor in series with a 2D MoS_2 FET. The equivalent capacitance model is shown in **Supplementary Figure 20**, with C_{CIPS}, the ferroelectric capacitance; $C_{h\text{-}BN}$, the dielectric capacitance; and C_{MoS_2}, the channel capacitance. The charge density of the CIPS capacitor (Q_{CIPS}) consists of a spontaneous polarization charge (P), and a paraelectric charge (Q_P)[16] which is written as

$$Q_{CIPS} = P + Q_P = P + C_{CIPS}V_{CIPS}$$

where C_{CIPS} is the linear capacitance of CIPS, and V_{CIPS} is the voltage drop across the CIPS capacitor when a top-gate voltage (V_{tg}) is applied. When V_{tg} goes to 0, one has

$$V_{tg} = V_{CIPS} + Q_{IS}/C_{IS} = 0$$

where Q_{IS} is the charge density of the underline MIS capacitor, and C_{IS} is its capacitance and is expressed as

$$C_{IS} = C_{h-BN}C_{MoS_2}/(C_{h-BN} + C_{MoS_2}).$$

Considering $Q_{IS} = Q_{CIPS}$, we obtain

$$Q_{CIPS} = \frac{PC_{IS}}{C_{IS} + C_{CIPS}}$$

so the depolarization field, or field in the ferroelectric CIPS, can be obtained by

$$E_d = \frac{V_{CIPS}}{t_{CIPS}} = \frac{Q_{CIPS} - P}{\varepsilon_{CIPS}} = -P[\varepsilon_{CIPS}(C_{IS}/C_{CIPS} + 1)]^{-1}$$

where t_{CIPS} and ε_{CIPS} are the thickness and dielectric constant of CIPS, respectively. The

negative sign in the above equation denotes that the depolarization field opposes the ferroelectric polarization.

Supplementary Note 3: Calculation of the retention time of a FeFET

As discussed in the main text, the retention loss is attributed to the depolarization field and charge trapping. When the retention time t→∞, total polarization that survives from back switching induced by the depolarization field (E_d) is expressed as[17]

$$P = P_r(1 - exp(-\frac{E_a}{E_d}))$$

where P_r is the remnant polarization, E_a is the activation field for polarization switching, and it is the same order of magnitude with a coercive field (E_c). Charges trapped in the gate dielectric stack also lead to a gradually diminished effect of polarization. The polarization loss per second due to charge trapping is estimated to be[18,19]

$$\Delta P \approx \sigma J N_{trap}$$

where σ is the effective trap capture cross section, J is the leakage current density, and N_{trap} is the trap density. Considering the contribution from both of the two mechanisms, the retention time of FeFET memory is estimated to be

$$t \approx \frac{P_r(1 - exp(-\frac{E_c}{E_d}))}{\sigma J N_{trap}}$$

The thickness of the ferroelectric layer is 50 nm for all FeFETs. The insulating layer for vdW FeFET is a 5-nm-thick h-BN, and for other FeFETs it is a 5-nm-thick SiO_2. The same leakage current density $J = 10^{-8}$ A/cm^2 and capture cross section $\sigma = 10^{-18}$ cm^2 were used to calculate retention time for all devices. Other parameters were obtained from the literature, as shown in **Supplementary Table 1**.

Reference

1. Lee, C. et al. Anomalous Lattice Vibrations of Single- and Few-Layer MoS_2. *ACS Nano* **4**, 2695-2700 (2010).
2. Park, B. E.; Ishiwara, H.; Okuyama, M.; Sakai, S.; Yoon, S. M., *Ferroelectric-Gate Field Effect Transistor Memories* (Springer, Netherlands, 2016).
3. Du, X., Zhao, M., Chen, G. & Zhang, X. Thickness Dependence of Ferroelectric Properties for Ferroelectric Random Access Memory Based on Poly(vinylidene fluoride-trifluoroethylene) Ultrathin Films. *Ferroelectrics* **488**, 148-153 (2015).
4. Laudari, A. & Guha, S. Polarization-induced transport in ferroelectric organic field-effect transistors. *J. Appl. Phys.* **117**, 105501 (2015).
5. Nugraha, M. I. et al. Broadening of Distribution of Trap States in PbS Quantum Dot Field-Effect Transistors with High-k Dielectrics. *ACS Appl. Mater. Inter.* **9**, 4719-4724 (2017).
6. Gong, N. & Ma, T. Why Is FE–HfO_2 More Suitable Than PZT or SBT for Scaled Nonvolatile 1-T Memory Cell? A Retention Perspective. *IEEE Electron. Dev. Lett*. **37**, 1123-1126 (2016).
7. Park, M. H. et al. Ferroelectricity and Antiferroelectricity of Doped Thin HfO_2-Based Films. *Adv. Mater.* **27**, 1811-1831 (2015).
8. Yurchuk, E. et al. Origin of the endurance degradation in the novel HfO_2-based 1T ferroelectric non-volatile memories. *2014 IEEE International Reliability Physics Symposium*, pp. 2E.5.1-2E.5.5 (2014).
9. Maikap, S. et al. Charge trapping characteristics of atomic-layer-deposited HfO_2

films with Al_2O_3 as a blocking oxide for high-density non-volatile memory device applications. *Semicond. Sci. Technol.* **22**, 884-889 (2007).

10. Maisonneuve, V., Cajipe, V. B., Simon, A., Von Der Muhll, R. & Ravez, J. Ferrielectric ordering in lamellar $CuInP_2S_6$. *Phys. Rev. B* **56**, 10860-10868 (1997).
11. Si, M. et al. Room-Temperature Electrocaloric Effect in Layered Ferroelectric $CuInP_2S_6$ for Solid-State Refrigeration. *ACS Nano* **13**, 8760-8765 (2019).
12. You, L. et al. Origin of giant negative piezoelectricity in a layered van der Waals ferroelectric. *Sci. Adv.* **5**, eaav3780 (2019).
13. Chen, L. et al. Thickness dependence of domain size in 2D ferroelectric $CuInP_2S_6$ nanoflakes. *AIP Adv.* **9**, 115211 (2019).
14. Vu, Q. A. et al. Near-zero hysteresis and near-ideal subthreshold swing in h-BN encapsulated single-layer MoS_2 field-effect transistors. *2D Mater.* **5**, 031001 (2018).
15. Lee, K., Liu, E.-S., Watanabe, K., Taniguchi, T. & Nah, J. Interface States in Bilayer Graphene Encapsulated by Hexagonal Boron Nitride. *ACS Appl. Mater. Inter.* **10**, 40985-40989 (2018).
16. Ni, K. et al. Critical Role of Interlayer in $Hf_{0.5}Zr_{0.5}O_2$ Ferroelectric FET Nonvolatile Memory Performance. *IEEE Trans. Electron. Dev.* **65**, 2461-2469 (2018).
17. Lou, X. J. Polarization retention on short, intermediate, and long time scales in ferroelectric thin films. *J. Appl. Phys.* **105**, 094107 (2009).
18. Bersuker, G. et al. Mechanism of Electron Trapping and Characteristics of Traps in HfO_2 Gate Stacks. *IEEE Trans. Device Mater. Rel.* **7**, 138-145 (2007).
19. Ma, T. P. & Jin-Ping, H. Why is nonvolatile ferroelectric memory field-effect

transistor still elusive? *IEEE Electron. Dev. Lett.* **23**, 386-388 (2002).
